# Launching the *Journal of Extracellular Biology* (J Ex Bio) – A new journal from the International Society for Extracellular Vesicles (ISEV)

**DOI:** 10.1002/jex2.19

**Published:** 2022-03-15

**Authors:** Andrew F. Hill, Susmita Sahoo

**Affiliations:** ^1^ Department of Biochemistry & Genetics, La Trobe Institute for Molecular Science La Trobe University Bundoora VIC Australia; ^2^ Cardiovascular Research Institute Icahn School of Medicine at Mount Sinai New York USA

The International Society of Extracellular Vesicles (ISEV) is proud to launch a new, open access journal called *the Journal of Extracellular Biology* (J Ex Bio), covering research on extracellular vesicles (EVs), extracellular particles (EPs), and related biology and applications.

With the incredible success of the *Journal of Extracellular Vesicles* (JEV), the ISEV Journal established in 2012, the Executive Board of ISEV began planning for a second journal in 2018. At the time, it was evident that the EV field was rapidly expanding and that the priority level for acceptance of articles into JEV was increasing. We felt that there was a clear need for another journal in the expanding field of EV research and related areas to fill in the knowledge gap from discoveries of newer types of particles and capture high‐quality science and widen the scope of topics covered in this emerging scientific area. In 2019, Kenneth Witwer and Marca Wauben developed a scoping document for what the ISEV Executive Board was calling ‘Journal 2’ at the time. This important document, written in the closing hours of the ISEV 2019 Annual Meeting in Kyoto, Japan, helped frame the development of the *Journal of Extracellular Biology*. Since then, much work has been undertaken by the ISEV Executive Board (including the Communications and Membership committee led by Susmita Sahoo) to further refine the scope of the journal and work with our publishing partner Wiley, and Talley Management Group to develop a business plan for the journal.

So, why the name ‘Extracellular Biology’? The journal is intended to be a vehicle for publishing EV research. Still, we felt there was scope to expand this to other EPs and entities interacting with, or involved in, EV biology. It is, however, increasingly appreciated that other EPs, many of which interact with and co‐fractionate with EVs, also contribute to their biological activities. With EVs now established as key players in intercellular communication and other biological functions, they can be harnessed as biomarkers and therapeutics. The overarching vision of J Ex Bio is to provide a broader forum to discuss EVs and their EP cousins: from biogenesis, on to interactions with each other and with other extracellular macromolecules (including but not limited to RNA, lipoproteins and protein complexes), extracellular matrix, and from there to ultimate effects on recipient cells and organs. J Ex Bio will promote interdisciplinary studies investigating the function of EVs and EPs in pathology, normal physiology, interorgan and interorganism communication, their cargo as biomarkers and therapeutics; new technologies and methods to engineer EVs and EPs and their artificial mimics.

The scope of J Ex Bio includes, but is not limited to, the following topics:

- Extracellular vesicles (all types of EVs) exosomes, microvesicles, ectosomes and others
- Other EPs released by cells, such as exomeres, RNA, lipoproteins (LDL, HDL, chylomicrons etc.) and protein complexes (e.g., ribonucleoproteins, nucleosomes), which may interact with EVs and EPs and may be involved in intercellular communication and biological functions
- Engineered EVs and EPs and their artificial mimics
- Research on their biology, biogenesis, diagnostics, therapeutics, methodology, technology and engineering
- Pathologies, physiologies, interorgan, inter‐organism communication
- Resource development, databases, analytical software, reagents, models and other tools
- Technical papers, negative results, confirmatory or contradictory results/studies

The topics covered by J Ex Bio are still broadly tied to EVs and could constitute the ‘future core’ of research that the current EV field is constantly exploring and expanding. Additionally, J Ex Bio will allow ISEV to focus on broader themes than those within JEV, yet align with ISEV's mission to ‘advance EV research globally’.

Key differences between the scope of J Ex Bio and JEV is that the scope of J Ex Bio is broader and will publish articles where EVs are not the primary focus, but other EPs are. Furthermore, J Ex Bio will also publish some of the products of ISEV Workshops and Taskforces where the topic meets the broader objectives of J Ex Bio.

J Ex Bio is now open for submissions. In early 2021, Andrew Hill was appointed Editor in Chief and an editorial board of global leaders in the areas in which J Ex Bio covers has been established. The three Deputy Editors are Juan M. Falcon‐Perez (Spain), Minh Le (Singapore) and Mỹ Mahoney (USA), who bring a wealth of scientific and editorial experience. The team of Associate Editors (see https://onlinelibrary.wiley.com/page/journal/27682811/homepage/editorial‐board for the list) also provide scientific leadership to the team.

We expect that J Ex Bio will become an important vehicle for publishing extracellular vesicle/particle research and contribute to the expanding scientific endeavours in this field, publishing original research articles, short reports, reviews and position papers. Fully open access, the journal will also provide wide reach for published articles. Article submissions can be made at the journal website (https://onlinelibrary.wiley.com/journal/27682811) which is available now.

